# An Exploration of How Solar Radiation Affects the Seasonal Variation of Human Mortality Rates and the Seasonal Variation in Some Other Common Disorders

**DOI:** 10.3390/nu14122519

**Published:** 2022-06-17

**Authors:** William B. Grant, Barbara J. Boucher

**Affiliations:** 1 Sunlight, Nutrition, and Health Research Center, P.O. Box 641603, San Francisco, CA 94164-1603, USA; 2 The Blizard Institute, Barts and The London School of Medicine and Dentistry, Queen Mary University of London, London E1 2AT, UK; bboucher@doctors.org.uk

**Keywords:** blood pressure, COVID-19, humidity, influenza, nitric oxide, respiratory infection, temperature, UVA, UVB, viral infection, vitamin D

## Abstract

Many diseases have large seasonal variations in which winter overall mortality rates are about 25% higher than in summer in mid-latitude countries, with cardiovascular diseases and respiratory infections and conditions accounting for most of the variation. Cancers, by contrast, do not usually have pronounced seasonal variations in incidence or mortality rates. This narrative review examines the epidemiological evidence for seasonal variations in blood pressure, cardiovascular disease rates and respiratory viral infections in relation to atmospheric temperature and humidity, and solar UV exposure through vitamin D production and increased blood concentrations of nitric oxide. However, additional mechanisms most likely exist by which solar radiation reduces the risk of seasonally varying diseases. Some studies have been reported with respect to temperature without considering solar UV doses, although studies regarding solar UV doses, such as for respiratory infections, often consider whether temperature can affect the findings. More research is indicated to evaluate the relative effects of temperature and sun exposure on the seasonality of mortality rates for several diseases. Since solar ultraviolet-B (UVB) doses decrease to vanishingly small values at higher latitudes in winter, the use of safe UVB lamps for indoor use in winter may warrant consideration.

## 1. Introduction

Several diseases have seasonal variations with the highest rates in winter and the lowest rates in summer.

**Figure 1:**
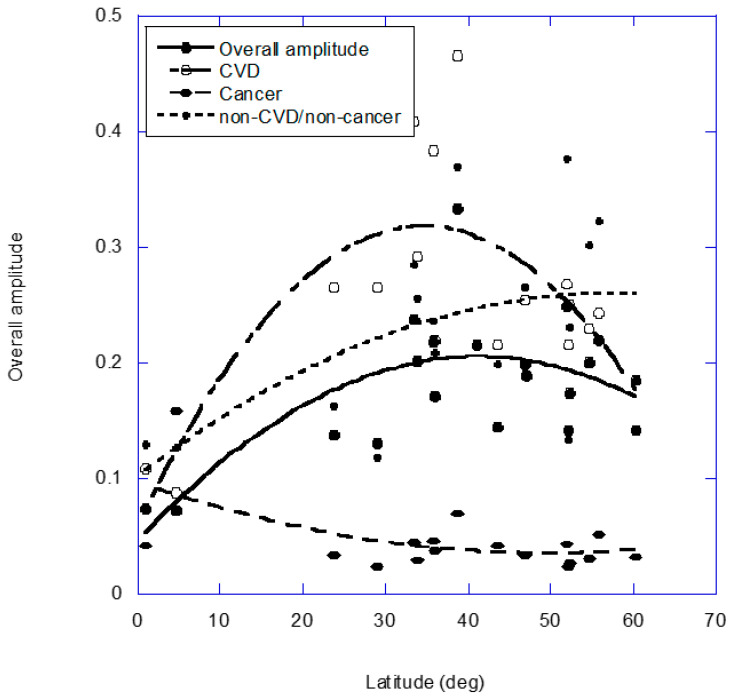
Plot of overall cardiovascular disease (CVD), cancer and non-CVD/non-cancer deaths showing seasonal variations versus ‘absolute’ latitude for 19 countries from Marti-Soler and colleagues [1]. The regression fits are second-order.

The seasonal variation in mortality rate is perhaps best illustrated by data assembled by Marti-Soler and colleagues [1]. Table 1 in [1] presents the seasonal variation (peak to nadir) in the ratio of observed to expected deaths for overall, cardiovascular disease (CVD), cancer and non-CVD/non-cancer for 19 countries ranging from Finland to New Zealand (60° N to 41° S). A plot of those data as a function of latitude is shown in Figure 1. CVD has an inverted U-shaped seasonal relation with respect to latitude, which is lower at both low and high latitudes than at mid-latitudes, whereas non-CVD/non-cancer death seasonal variations increase with respect to latitude, and cancer seasonal rates are higher inside than outside the tropics.

**Figure 2:**
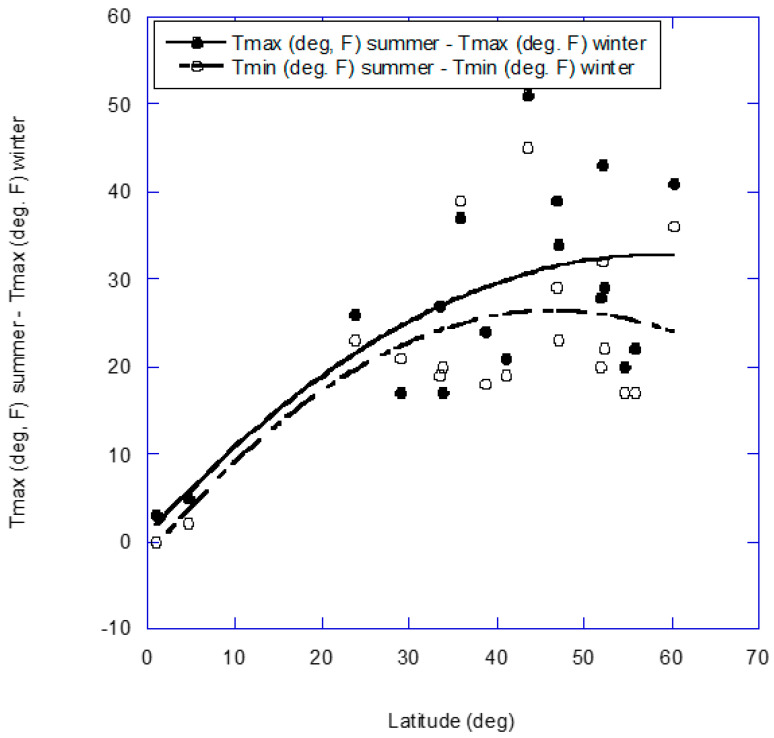
Seasonal variation in high minus low ambient atmospheric temperature for the approximate center of population for 18 countries from Marti-Soler and colleagues [1]. The regression fits are second-order.

**Figure 3:**
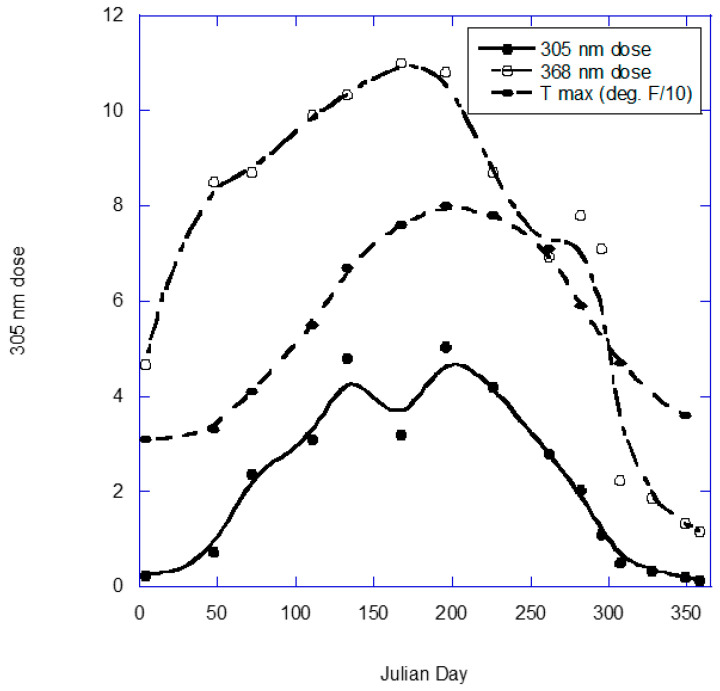
Annual variation for solar UVA and UVB in Geneva, NY, USA (42.9° N) for July 2006 to June 2007, as well as the temperature for 2021. UV data are from the UV-B Monitoring and Research Program operated by Colorado State University for the U.S. Department of Agriculture, https://uvb.nrel.colostate.edu/UVB/da_queryLampIrradiance.jsf (accessed on 1 June 2022). Temperature data are from https://www.usclimatedata.com/climate/geneva/new-york/united-states/usny0548 (accessed on 1 June 2022).

Several factors affect the risk of at least one of these disease categories and vary with latitude. Those factors include ambient atmospheric temperature [2,3], solar ultraviolet-B (UVB) production of vitamin D [4,5,6] and solar UVA induction of nitric oxide (NO) in the serum [7,8,9]. Figure 2 shows the seasonal variation in high minus low ambient atmospheric temperature for the approximate center of population for 18 countries from reference [1] (USA was omitted), and Figure 3 shows the seasonal variations in solar UVA, UVB and temperature for Geneva, NY, USA (https://uvb.nrel.colostate.edu/UVB/uvb-network.jsf, accessed on 1 June 2022).

A book chapter [10] reported that, in Europe, from Greece (38° N) to Iceland (64° N), a U-shaped relationship exists between mean country serum 25-hydroxyvitamin D [25(OH)D] for adults in winter with higher concentrations at lower and higher latitudes than intermediate latitudes; however, mean concentrations in summer were near 68 nmol/L. The likely reasons for the inverted U-shape include that, at lower latitudes, solar UVB lasts longer than at higher latitudes [11], whereas at higher latitudes, national diets have more cold-water ocean fish and meat [12]. In addition, inhabitants in northern countries are more likely to take vitamin D supplements.

An article by Liu and colleagues [8] reported laboratory studies showing that UVA irradiation of the skin lowered BP, that UVA increases nitrite and reduces circulating nitrate, and that UVA increase forearm blood flow. NO is released by nitrite photolysis to nitrate. They predicted NO release from stores in the skin due to UV exposure as a function of latitude for summer and winter. The peak wavelength for the action spectrum was 330 nm, and it was estimated that the UVA spectrum (315–400 nm) is responsible for 80% of the release. The winter to summer difference in NO release rate peaks between 40° and 60° which correlates with the observed seasonal variation in BP and CVD rates and the summertime NO release rate decreases rapidly with increasing latitude in summer, which mirrors the relationship of blood pressure (BP) and hypertension prevalence. 

**Figure 4:**
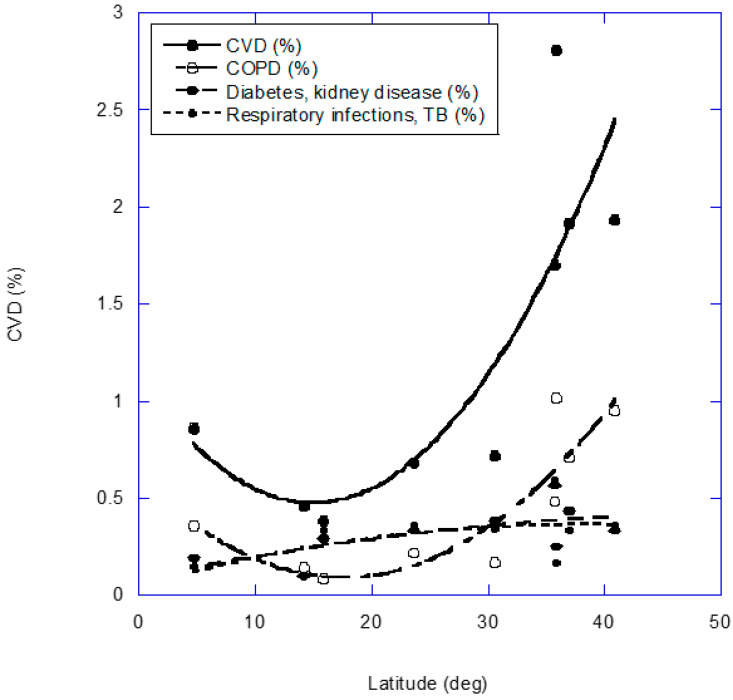
Percentage of deaths attributable to low temperatures for cardiovascular disease (CVD), chronic obstructive pulmonary disease (COPD), diabetes, kidney disease, respiratory infections and tuberculosis (TB) versus absolute latitude (doi:10.1016/S0140-6736(21)01700-1) [3]. The regression fits are second-order.

The importance of temperature is underscored from analyses of the dependence of mortality rates when related to ambient temperature. Analyses based on 64.9 million deaths from 9 countries between 1 January 1980 and 31 December 2016, for 17 causes of death, were reported in 2021 [3]. Of particular interest for the present study was the finding that cold-related increased mortality rates are driven largely by CVD, chronic respiratory disease, metabolic disease and acute respiratory infections. Figure 4 shows the geographical variation in the percentage of deaths attributable to low temperatures for CVD, COPD, diabetes and kidney disease, respiratory infections and tuberculosis (TB) [3]. Only CVD as well as respiratory infections and TB have significantly higher percentages of deaths attributable to cold temperatures and higher latitudes.

Solar UVB production of vitamin D has been linked to seasonal variations in several diseases. In 1981, Scragg proposed a mechanism to explain CVD’s seasonal variations [13]. Rostand added blood BP in 1997 [14]. In 2002, Ponsonby and colleagues added three autoimmune diseases: multiple sclerosis, type one diabetes and rheumatoid arthritis [15]. In 2006, Cannell and colleagues proposed adding epidemic influenza [16]. In 2010, Lindqvist and colleagues added type two diabetes mellitus and metabolic control [17]. Studies in Sweden reported that avoiding sun exposure significantly increases mortality rates for women [18,19,20]. A review of mortality rates in the United States as a function of the day of the year suggested that seasonal variations in serum 25(OH)D concentrations are the primary driver of seasonal variations of mortality rates, although temperature variations may also play a role [6].

Notably, because the 25(OH)D action spectrum is in the solar ultraviolet-B (UVB) range (290–315 nm), vitamin D cannot be produced from solar radiation at high latitudes in winter. The classical study was reported by Webb and Holick in 1988 [21]. They showed that producing vitamin D from sun exposure is not possible for the darkest 6 months of the year in Boston (42.4° N) and Edmonton (53.5° N). Engelsen showed the time necessary to produce 400 IU of vitamin D for a young person with his or her face, neck and hands exposed as a function of latitude and time of the year to be 15 min [11]. Winter 25(OH)D concentrations are generally maintained at about 50–70% of summertime concentrations in Europe and the United States [22,23]. Vitamin D, as 25(OH)D, is recycled from the skeletal muscles by the action of parathyroid hormone (PTH) [24,25].

NO’s role in human health was the subject of intense research starting in about 1977, with work by Murad and colleagues showing the stimulation of guanylyl cyclase by NO [26]. Stimulators of guanylate cyclase have been used to treat heart failure and pulmonary arterial hypertension. In 1979, Ignarro and colleagues [27] showed that NO was important in activating coronary arterial wall cell guanylyl cyclase. Furchgott and Zawadzki [28] showed that NO was involved in acetylcholine-induced relaxation and constriction of arterial walls through effects on the smooth muscle they contain. All three were awarded the Nobel Prize in 1998 [29]. A 20-year review of their work was published in 2019 [30].

The first review of NO produced in the skin through UV exposure was published in 1997 [31]. The proposed mechanism of production was the upregulation of the gene for inducible NO synthase (iNOS) enzyme. The benefits of NO mentioned included regulating blood flow, wound healing, preventing infections and improving eczema and psoriasis. In 2009, Oplander and colleagues reported that whole-body UVA exposure lowered BP by releasing NO from intracutaneous photolabile NO derivates [32]. Further early reviews of UV production of NO and its health benefits were published in 2011 by Juzeniene and colleagues [33] as well as by Juzeniene and Moan [34]. Later, Weller and colleagues explored the role of UV-induced NO production for various health outcomes, including lowering BP [8], improved physical performance [35], reducing liver inflammation [36] and reducing metabolic dysfunction experimentally [37].

The primary goal of the present report is to examine the roles of factors related to solar radiation that can affect the risk and seasonality of the three major diseases exhibiting pronounced seasonality of mortality: CVD, hypertension and respiratory tract infectious diseases. A secondary goal is to identify, and briefly discuss, other diseases and adverse health outcomes with seasonal variations, where the highest rates are seen in winter.

For this narrative review, literature searches were conducted with Google Scholar (scholar.google.com) and the National Library of Medicine’s PubMed database (pubmed.gov). Searches were conducted for the seasonality of mortality and seasonality of other health outcomes. The primary factors investigated are the potential roles of solar UVB, vitamin D, solar UVA, NO and ambient temperature in explaining the observed seasonal variations. The search term “countries” was included in some of the searches in order to find multi-country studies of mortality rates and temperature effects on mortality rates. When appropriate multi-country studies were found, such as for seasonal mortality rates for the major diseases reviewed in this work, single-country studies were omitted. In addition, information on other factors that may affect seasonality was considered, including seasonal variations in gene expression and the effect of air pollution on disease incidence and mortality rates. Searches were also conducted regarding UVB lamps for indoor use with humans. Appropriate sources were found for data required regarding the seasonal variation of solar UVA and UVB doses, COVID-19 case rates, winter and summer temperatures of countries and latitudes of countries.

## 2. Results

### 2.1. Blood Pressure

Several environmentally related factors affect BP. This review considers temperature, vitamin D, sunlight and NO.

An analysis of the data of 38,589 participants in Harbin from the China Kadoorie Biobank during 2004–2008 reported an increase of 6.7 mmHg in systolic BP (SBP) and 2.1 mmHg in diastolic BP (DBP) for each 10 °C decrease in outdoor temperature when outdoor temperature was above 5 °C. An inverse association was evident between outdoor temperature and cardio-cerebrovascular event morbidity [38]. A study from Guangdong Province, China, also reported reductions in BP with increasing ambient temperature up to 20 °C, as well as decreased rates of hypertension prevalence [39].

Rostand first reported latitudinal variations in BP and hypertension [14]. Using data from the INTERSALT study [40], he showed that a linear regression fit to SBP increased from 108 mmHg (95% confidence interval [CI], 104–113 mmHg) at the equator to 125 mmHg (95% CI, 121–129 mmHg) at 70° from the equator. Diastolic BP (DBP) increased from 67 mmHg (95% CI, 63–70 mmHg) at the equator to 78 mmHg (95% CI, 76–81 mmHg) at 70° from the equator. Using both INTERSALT and non-INTERSALT data, he found that hypertension rates increased from 8% (95% CI, 3–13%) at the equator to 25% (95% CI, 20–29%). He proposed that UVB, through effects on both circulating 25(OH)D and parathyroid hormone [PTH] concentrations as well as in part through affecting intracellular calcium, may explain the findings.

Rostand and colleagues [41] reported data on BP, sunlight and 25(OH)D concentrations among black and white participants in the Reasons for Geographic and Racial Differences in Stroke (REGARDS) Study conducted in the stroke belt states of the southeastern United States [41]. These researchers found inverse correlations between solar radiation and SBP for white people but not black people, and for women but not men. The group found no evidence that 25(OH)D concentrations are related to SBP. They then cited the possible role of UVA in increasing serum NO concentrations [8,32,42,43].

In 2020, Weller and colleagues published an article and correspondence regarding the role of UV exposure and changes in BP [44,45]. The article reported the results of a study of 342,459 patients (36% black, 64% white) at 2178 dialysis centers over 3 years. The researchers used incident UVA, UVB and temperature data from the National Oceanic and Atmospheric Administration database. They found linear inverse correlations between SBP and UVA, UVB and temperature. Although the BP findings for UVA and UVB were reduced when temperature was added to the analysis (by 57% for UVA and by 56% for UVB), the reductions with higher UVA and UVB were still significant. Predialysis SBP was about 4 mmHg higher for black patients. The correspondence reported that UVB’s effect on BP was stronger than that of UVA. The authors argued that, because the evidence at that time for vitamin D in regulating BP was weak to nonexistent, the effect was due to increases in NO. However, that assumption has been overturned by a recent Mendelian randomization study that stratified the analysis by a genetically inferred 25(OH)D concentration [46]. In addition, supplementation by high-dose vitamin D_3_ supplementation (~4000 IU/d) in an open label observational study in Canada that increased 25(OH)D concentrations above 100 nmol/L was found to reduce SBP by 14 to 18 mmHg and DBP by ~12 mmHg for hypertensives; however, no significant reduction in BP was found for pre-hypertensive participants. [47]. In addition, 71% of the 592 patients who were hypertensive at the beginning of te study were no longer hypertensive. 

In summary, higher ambient temperatures (up to about 20 °C), higher NO concentrations, and higher 25(OH)D concentrations, i.e., higher vitamin D status, do appear to reduce BP. More research is indicated to assess each factor’s relative contribution.

### 2.2. All-Cause Mortality Rate

Ambient temperature’s effect on mortality rates has been known for many years. The historical review and analysis by Kutschenreuter [48] is a good source of information from the USA and serves as a good starting point. Figure 1 in that article uses monthly temperature data from 1921 to 1950 and monthly all-cause mortality rate data from 1949 to 1958 for three U.S. cities: Cincinnati, OH, USA; Los Angeles, CA, USA; and New York, NY, USA. The curves for all three cities show strong inverse correlations between ambient temperature and mortality rate. The amplitudes of mortality rate are +23% for a 46 °F change in temperature in Cincinnati, +25% for a 18 °F change in temperature in Los Angeles and +27% for a 44 °F change in temperature in New York. Figure 2 shows that the percent seasonal amplitude for all-cause mortality is in the low 30s for people older than 25 years but very low for those aged 1–24 years. Figure 4 shows that the seasonal variation in mortality rates is similar for white people and non-white people. All those results are consistent with the idea that changes in ambient temperature strongly influence mortality rates.

The article regarding the role of non-optimal temperature on daily mortality by Marti-Soler and colleagues [1] is very useful. Figure 1 in that article shows that, for lower respiratory infections, ischemic heart disease, stroke and chronic obstructive pulmonary disease, mortality increases for temperatures both below or above the theoretical minimum-risk exposure level (TMREL), which was calculated for temperatures from 6 °C to 28 °C. Evidently, the TMREL varies according to the mean temperature of the location. In a table, they estimated the attributable deaths and population attributable fractions (PAFs) for high and low temperatures for nine countries. Representative PAFs for low temperature in 2019 were 1.08% (95% CI, 0.92–1.31%) for Guatemala, 3.44% (95% CI, 3.03–3.75%) for the USA and 4.28% (3.88–4.66%) for China.

A study in the UK reported that winter mortality was explained almost entirely by a combination of monthly average temperatures over the previous 12 days, and weekly influenza A counts [49].

The role of UVB and vitamin D in reducing all-cause mortality rates is also well-known. For example, an observational study in Sweden involving 20,518 women aged 25–64 years recruited from 1990 to 1992 and followed up for 20 years reported that sun avoiders, in comparison with people with the highest sun exposure, had their life expectancy reduced by 0.6–2.1 years [19]. A pooled analysis of 32 observational studies showed that the hazard ratio for death decreased by 1.9 (95% CI, 1.6–2.2) for 25(OH)D < 10 ng/mL compared to 25(OH)D > 30 [50].

### 2.3. Cardiovascular Disease

Temperature has an important impact on CVD risk. In a study from Moscow, Russia, researchers found a relationship between average daily temperature and CVD mortality rates [51]. A plot of the basic mortality–temperature relationship indicated that this relationship was V-shaped with the minimum at ~18 °C. Each 1 °C increment of average daily temperature above 18 °C resulted in an increase in deaths from coronary heart disease (CHD) by 2.7%, from CVD by 4.7% and from respiratory diseases by 8.7%, with a lag of 0 to 1 day. Each 1 °C drop of average daily temperature from 18 °C to –10 °C resulted in an increase in deaths from CHD by 0.57%, from cerebrovascular diseases by 0.78%, and from respiratory diseases by 1.5%, with lags of maximum association varying from 3 days for non-accidental mortality to 6 days for cerebrovascular mortality.

Another article has reported exposure–response curves for ischemic heart disease and stroke [3], and it showed that the temperature effect depends on the daily mean temperature of residence, with people adapted for living with mean temperatures above 20 °C having reduced risks of mortality at up to 28 °C as compared to those of people living where mean temperatures were below 20 °C. The attributable percentage contribution of temperature to CVD mortality rate near 40° is 2.5%. A seasonal variation of 0.3 near 40° implies that the mean increase is near 15%. Thus, 17% (2.5/15) of the seasonal variability can be attributed to cold temperature. However, reducing the risk of CVD due to low temperatures is clearly possible by wearing warm clothing when outdoors in winter [52] or by staying in a heated home or work environment [53]. Similar results for CVD mortality rates with respect to temperature were also reported for residents of Spain [54]. The RR of CVD deaths was higher at 30 °C than at –5 °C, and the effect of low and high temperatures decreased from 1980–1994 to 2002–2016. The overall (all ages) RR at –5 °C compared with 20 °C was 1.5 in 2002–2016, whereas that for 30 °C was 1.8.

An important mechanism to explain why exposure to cold temperatures increases the risk of CVD mortality rates comes from Keatinge [55]. The body responds to cold by shifting up to a liter of blood from the skin to the internal organs to reduce heat loss. This shift leads to disposal of the extra fluid and salt, partly by the kidneys as urine and partly into the body’s general intercellular spaces, thereby increasing blood viscosity and increasing the risks of thrombosis [55]. The effect of high temperature on risk of CVD mortality appears to relate to the effects of aging on thermoregulation during heat stress [56]. Compared with young adults during heat stress, older individuals typically respond with less individual sweat gland output, decreased skin blood flow, reduced cardiac output and smaller redistributions of blood flow from the splanchnic and renal circulations.

CHD rates vary with respect to the latitude of countries and season [57,58]. However, the geographical variation in data on a smaller scale, such as within a country, correlated most strongly with small particulate aerosol (PM_2.5_) concentrations, as found in the United States [59].

Observational studies have shown significant inverse correlations between CVD incidence and 25(OH)D concentrations [46]. In addition, an observational study based on patients of the U.S. Veterans Health Administration system with a baseline serum 25(OH)D concentration of <20 ng/mL who were counseled to take vitamin D supplements to raised serum 25(OH)D to >30 ng/mL had a 35% (95% CI, 15–51%) reduced risk of myocardial infarction (MI) compared with those who remained at <20 ng/mL [60]. However, clinical trials have consistently failed to show that vitamin D supplementation reduces CVD incidence [61,62], casting doubt on findings from observational studies. By contrast, a randomized controlled trial (RCT) showed that high-dose vitamin D_3_ supplementation reduces arterial stiffness in overweight African Americans [63].

In addition, two Mendelian randomization (MR) studies reported inverse correlations between genetic predictions of 25(OH)D concentrations by using data from the UK Biobank. Using nonlinear analysis, one study showed that CVD risk started to rise with lower genetic 25(OH)D concentrations in subjects with 25(OH)D concentrations below 20 ng/mL, progressing to a rapid increase in those with 25(OH)D values below 10 ng/mL [46]. The odds ratio (OR) for CVD for a 25(OH)D level of 10 ng/mL compared with 30 ng/mL was 1.11 (95% CI, 1.05–1.18). A similar relationship was also found for SBP. Thus, the authors estimate that approximately 6% of CVD risk in the UK can be prevented by raising serum 25(OH)D concentrations to above 40 ng/mL.

The second study used a stratified (nonlinear) analysis with respect to residual 25(OH)D concentrations [64] (calculated as the residual from the regression of seasonally adjusted 25(OH)D on the mean-centered generic risk score), which allowed for the comparison of individuals who would have had 25(OH)D concentrations in the same stratum if they had the same genotype. CVD mortality (6150 events) was significantly reduced in that MR analysis for 25(OH)D values of < 10 ng/mL by genotypes known to raise serum 25(OH)D (estimate = 0.69 [95% CI, 0.52–0.92]; *p* = 0.01). As noted in that article, MR uses genetic variants specifically related to a particular exposure to compare genetically defined population subgroups with different average levels of the exposure. The independent segregation of alleles at conception means that these genetically defined subgroups should not differ systematically with respect to confounding variables, creating a natural experiment analogous to that of a randomized trial. Thus, these two MR studies offer evidence that low 25(OH)D concentrations are significantly causal for CVD risk. Unfortunately, vitamin D RCTs have not yet been designed to be able to investigate this effect adequately [65,66].

Further investigation shows that high PTH values associated with low 25(OH)D concentrations, especially in the elderly, play an important role in CVD risk. A study in Utah reported that higher PTH at the baseline was noted in 26.1% of the study population. Highly significant differential CVD prevalence/incidence rates for most CVD risk factors, disease diagnoses and mortality were noted for PTH > 75 pg/mL (by 1.25- to 3-fold). PTH correlated only weakly with 25(OH)D and moderately with glomerular filtration rates. Risks related to PTH were attenuated slightly after adjusting for confounding factors, but they stayed significant [67].

An analysis of measurements of PTH and 25(OH)D in >300,000 U.S. patients confirmed that PTH increases with age and is inversely correlated with 25(OH)D concentrations. Patients most likely to have PTH > 75 pg/mL were older than 60 years and had 25(OH)D < 10 ng/mL [68]. PTH stimulates aldosterone secretion by increasing calcium concentrations in the cells of the adrenal zona glomerulosa as a result of binding to the PTH/PTH-rP receptor and indirectly by potentiating angiotensin-two-induced effects [69]. Meta-analyses of calcium supplementation studies reported significant increases in the risk of myocardial infarction compared with placebo and nonsignificant increases in the risk of stroke [70], which were not seen with comparable increases in dietary intakes [71].

A study of the seasonal variation of 25(OH)D concentrations in British adults aged 45 years indicated that 16% had values below 10 ng/mL in winter compared with 3% in summer [22]. Thus, this seasonal variation in vitamin D status appears large enough to contribute, in part, to the seasonal variation of CVD through a PTH-related mechanism.

A 2013 review by Lei and colleagues summarized the mechanisms by which NO reduces the risk of CVD. NO inhibits smooth muscle cell proliferation and migration; enhances the proliferation and migration of endothelial cells and inhibits their apoptosis; suppresses platelet aggregation; and prevents platelet, leukocyte and monocyte adhesion to endothelium. NO also inhibits the development of intimal hyperplasia in mechanically or immunologically injured vessels [72]. In 2016, Weller outlined the evidence that UVA-stimulated increase in levels of NO in the blood can play a role in reducing risk of CVD [73]. The hypothesis is supported by hypertension’s importance as a risk factor for CVD [74]. 

A study in Scotland offered more support for UVA in reducing risk of MI [75]. A total of 56,370 MI patients were followed up from 2000 to 2011. Solar UVA and UVB doses were obtained from NASA satellite instruments. Monthly acute MI hospital admissions ranged from 6 to 11 out of a population of 100,000 during that period, whereas seasonal variations were about 1.5/100,000. The seasonal variations superimposed on the underlying trend had an amplitude of 0.31/100,000 (95% CI, 0.21–0.41/100,000). The log of UVA was significantly correlated with the amplitude after adjusting for UVB, with a correlation coefficient of –0.08 (95% CI, –0.13 to –0.02; *p* = 0.008). That amplitude was not significantly correlated with UVB nor temperature. Since the seasonal variations were small, UVA did not seem to have a large impact on seasonality.

A paper published in 2017 reviewed the environmental determinants of CVD [76]. The topics considered that could affect seasonality directly were discussed in this order: temperature, solar UVB and vitamin D, then solar UVA and NO. In addition, several other factors that affect CVD risk were discussed, of which air pollution and physical inactivity did impact the risk of CVD, with air pollution likely to be more important in the summer and physical inactivity more important in the winter.

In summary, temperature appears to be the most important factor regulating seasonal variations in CVD mortality rates. Temperature has the strongest support with respect to both short-term and seasonal variations in temperature [3]. However, the effect of 25(OH)D concentrations mediated by PTH and calcium is also important at older ages. UVA-stimulated release of NO is most likely important in reducing the risk of CVD death, but more research is required. More research is also indicated to better determine the relative strength of the effects of these factors on the risk of death from CVD.

### 2.4. Viral Infectious Diseases of the Respiratory Tract

Epidemic influenza has large seasonal variations, with peak rates 6 months apart in the northern and southern hemispheres [77]. Cannell proposed that solar UVB, through the production of vitamin D, may explain this epidemiology [16]. This hypothesis is supported by some clinical trials, such as one involving African American women in New York with a mean 25(OH)D baseline of 19 ± 8 ng/mL [78]. However, the hypothesis is not supported in another trial with African American women with a mean 25(OH)D baseline of 26 ± 12 ng/mL [79], where fewer cases of vitamin D deficiency must have been present. Support was also found for vitamin D supplementation in reducing the risk of influenza A but not influenza B in a study involving schoolchildren in Japan [80]. However, an analysis of seasonal influenza data from the Health Professionals Follow-up Study showed that absolute humidity and the school calendar better explained the seasonal patterns than did seasonal variations in 25(OH)D concentrations [81]. A later article reported that, where monthly average specific humidity or temperature decreased below thresholds of approximately 11–12 g/kg and 18 °C–21 °C during the year, influenza activity peaked during the cold–dry season (i.e., winter) when specific humidity and temperature are at minimal levels [82]. For tropical regions, seasonal influenza tends to peak in seasons of rainfall greater than 150 mm/month.

An observational study was conducted of the temporal variation in influenza and meteorological variables in several Northern European countries from 1 September 2017 to 31 August 2018 [83]. Temperature had the highest correlation with influenza, followed by the UV index, humidity, wind speed, precipitation and atmospheric temperature. The peak influenza rate occurred in calendar week 7 (i.e., mid-February), with only 10% of the peak rate seen in weeks 50 and 17. According to an analysis of solar UVB doses as a function of latitude and time of year, producing vitamin D from winter solar UVB is virtually impossible above 55° N from about week 53 to week 6 [11]. Similarly, a study of 45-year-old inhabitants of Great Britain from September 2002 to March 2004 reported that 25(OH)D concentrations were minimal in winter, with little change from January through April [22]. Thus, the UV effect on influenza rates observed was not due to UVB exposure or vitamin D production, suggesting that the UV effect was due to UVA rather than to UVB—something Cannell and colleagues did not evaluate [16].

COVID-19 rates also vary seasonally, with higher rates in winter but with occasional outbreaks in summer when a new variant of the SARS-CoV-2 virus arises. An analysis of the seasonality of viral infections noted that they spread faster in temperate regions when it is cold and dry, since the viruses remain viable longer ex vivo in such conditions [84,85]. However, in tropical regions, higher transmission is better supported by higher humidity, since the stability of the viruses ex vivo is maintained in droplets and on moister surfaces. Cold, dry conditions also reduce the body’s immune response to virus infections by compromising nasal muco-ciliary clearance and local immune responses [86].

A recent article reported the correlations between meteorological and air quality variables and COVID-19 case rates in Morocco between 2 March and 31 December 2020 [87]. The factors that were associated with increased risk were relative humidity above 80%, wind speed above 20 m/s, ozone concentrations above 5 µg/m^3^ and PM_10_ concentrations above 120 µg/m^3^. Factors associated with lower risk were temperatures above 25 °C, precipitation above 25 mm and insolation above 10 h/day.

Grant and colleagues proposed in April 2020 that vitamin D reduces the risk of SARS-CoV-2 infection and COVID-19 [88]. Many studies have examined that hypothesis. The strongest support comes from two observational studies of SARS-CoV-2 infection or COVID-19 outcomes with respect to vitamin D supplementation. One prospective study conducted in Spain showed that patients prescribed with cholecalciferol or calcidiol and achieved 25(OH)D >30 ng/mL compared with untreated controls with 25(OH)D <20 ng/mL, had a significantly reduced risk of SARS-CoV-2 infection (multivariate analysis hazard ratio (maHR) = 0.66 [95% CI, 0.57–0.77; *p* < 0.001]) and COVID-19 mortality (maHR = 0.66 [95% CI, 0.46–0.93; *p* < 0.001]) and a significantly reduced risk of severe COVID-19 (maHR = 0.72 [95% CI, 0.52–1.00; *p* = 0.05]) [89]. Similar results were found for calcifediol prescriptions, with *p* ≤ 0.001 for reductions in the risk of catching COVID-19 and in its severity and mortality.

The second observational study involved veteran patients receiving care at the U.S. Department of Veteran Affairs health care facilities between 20 February and 8 November 2020 [90]. Serum 25(OH)D data measured within 15–90 days before a positive SARS-CoV-2 test were available for 4599 patients. The adjusted probability of hospitalization fell from 0.25 ± 0.03 at 13 ng/mL to 0.18 ± 0.02 at 60 ng/mL. The adjusted probability of mortality fell from 0.11 ± 0.02 at 13 ng/mL to 0.06 ± 0.01 at 60 ng/mL. Since 60 ng/mL is higher than the likely mean 25(OH)D of ~35 ng/mL that the patients could have achieved from solar UVB exposure, the higher 25(OH)D concentrations most likely represent the result of vitamin D supplementation.

In an observational study in Spain, treating COVID-19 patients with calcifediol significantly reduced mortality rates [91]. The trial included 76 consecutive COVID-19 patients in a Cordoba hospital. A total of 50 patients were treated with high-dose calcifediol [25(OH)D_3_], which has an advantage over vitamin D of rapidly increasing serum 25(OH)D concentrations. The multivariate risk estimate [OR] for admission to the intensive care unit for calcifediol treatment plus the conventional treatment versus the conventional treatment alone was 0.03 (95% CI, 0.003–0.25), and no patients treated with calcifediol died, whereas 2 not treated with calcifediol died.

A recent RCT involving health care workers in four Mexican hospitals reported that taking 4000 IU/d of vitamin D_3_ for 1 month reduced SARS-CoV-2 infection rates by 75% [RR = 0.23 (95% CI, 0.09–0.55)] [92]. The effect was related to an increase in median 25(OH)D concentrations from 18 to 27 ng/mL

Chronic obstructive pulmonary disease (COPD) also has significant seasonal variations of exacerbations and mortality rates. The Towards a Revolution in COPD Health (TORCH) study involved 6112 COPD patients from 42 countries followed up for 3 years [93]. The patients were treated with standard drugs or placebo and visited clinics at 12-week intervals. In the northern hemisphere, exacerbations were 9.3% in winter and 5.3% in summer, whereas in the southern hemisphere, exacerbations were 12% in winter and 6% in summer. Findings of a review published in 2014 suggest that this seasonality was partly due to the increased prevalence of respiratory viral infections circulating in cold, damp conditions, along with increased airway inflammation or reduced 25(OH)D concentrations [94]. Vitamin D reduces inflammation through effects on cytokine production [95].

Evidence also exists that supports the role of UVA-induced increases in serum NO concentrations in reducing the risk of SARS-CoV-2 infection and COVID-19. An analysis of the geographical variations of COVID-19 mortality rates in England, Italy and the United States from January to April 2020 reported significant inverse correlations with respect to solar UVA doses in regions of each country when solar UVB doses were zero [9]. The adjusted mortality risk ratios per 100 kJ/m^2^ increase in mean daily UVA were 0.49 (95% CI, 0.38–0.64) in England, 0.81 (95% CI, 0.71–0.93) in Italy and 0.71 (95% CI, 0.69–0.85) in the U.S. The pooled estimate was 0.68 (95% CI, 0.52–0.88).

Another study was conducted within Harvard’s Nurses’ Health Stufy II involving 39,315 participants, with 1768 testing positive for SARS-CoV-2 between May 2020 and March 2021 [96]. Higher predicted 25(OH)D concentrations, but not vitamin D intake, were associated with reduced SARS-CoV-2 infection rates. The highest quartiles of both UVB (annual) and UVA (winter) doses at residence locations were associated with a ~24% reduction in infection rates compared with the lowest doses.

A recent article reported that narrowband UVB (nUVB) treatment is very beneficial in treating hospitalized COVID-19 patients [97]. The study involved 30 COVID-19 patients in Louisiana in mid-2021, of whom 15 were randomized to be treated with nUVB and 15 with the same lamp but with the UVB blocked with UV-absorbing plexiglass. Patients were treated for up to 8 days. The end result was that two nUVB-treated patients died within 28 days compared to five untreated patients (*p* = 0.39). Interestingly, 25(OH)D concentrations on day 5 had decreased by −12 ng/mL (95% CI, −20 to −5 ng/mL) versus +1.7 ng/mL (95% CI, −12.1 to 7 ng/mL). The authors suggested that vitamin D was consumed by an nUVB-driven response to COVID-19. Although the primary endpoint was not significant, this study provided reasonable evidence that nUVB treatment effects were due to non-vitamin D effects. This concept is supported by Richard Weller, Centre for Inflammation Research, University of Edinburgh, Edinburgh, UK (private communication, June, 2022).

An article published in 2021 examined the surge dates for COVID-19 (based on SARS-CoV-2 seropositive case rates) in European countries during autumn of 2020 [98]. In Figure 3 of that article, the surge dates range from 10 September for Iceland to 22 October for Greece. The linear regression fit to the data has an *r*^2^ value of 0.77. Figure 4 in that article shows that the surge date corresponds to solar UVB dose between 30% and 40% of the maximum for day 182 in July. A problem with attributing the effect to vitamin D is that serum 25(OH)D concentrations peak near 75 nmol/L in the UK (latitude, 52° N) in September and then decrease at a rate of 6–7 nmol/L to February or March [22]. Thus, the effect of vitamin D was too small and slow to explain the finding. A better explanation is that solar UVB induces various biological effects, both known and unknown, that reduce the risk of SARS-CoV-2 infection and COVID-19 [99], while UVA could also play a role.

In the winter in non-tropical regions, increases in solar UVA increases and in serum NO, atmospheric temperature and humidity appear to have the greatest impact on influenza and COVID-19 disease rates. In the summer, the non-vitamin D effects of UVB exposure and temperature/humidity seem to predominate, with some benefit from vitamin D.

### 2.5. Cancer

As reported by Marti-Soler and colleagues [1], cancer mortality rates do not have significant seasonal variations. Unlike CVD and respiratory tract infections, cancer mortality rates have significant inverse correlations with solar UVB in ecological studies [100]. Clinical trials have confirmed that vitamin D supplementation reduces the risk of cancer mortality [101]. Ambient temperature does not seem to affect cancer incidence or mortality rates. Two ecological studies, one in the entire U.S. [102] and one in Florida [37], showed an increase in cancer rates at colder temperatures. However, the studies did not consider solar UVB doses, which likely explain the findings. A recent review outlined the case for cold temperature increasing the risk of cancer [103]. Endothelial iNOS is a risk factor for several cancers, such as breast cancer [104], prostate cancer [105] and gastric cancer [106]. Since endothelial iNOS increases NO concentrations, these findings do not support UVA-induced increases in serum NO as reducing the risk of cancer.

Vitamin D deficiency is associated with increased colorectal cancer risks that appear likely to be causal [100]. Breast cancer survival is increased in people diagnosed in summer or autumn rather than in winter, although whether that is influenced by temperature, by vitamin D or by hormonal factors is unclear [107,108]. Vitamin D supplementation reduced cancer mortality though not cancer incidence in the VITAL study [109]. Data for the effects of NO, UVB or UVA are lacking, although mortality is reduced with greater sun exposure in Norway for several cancers, including breast, lung and prostate tumors [108]. Vitamin D supplementation has improved survival in breast cancer [110], but specific data for the effects of NO or UV are not available.

### 2.6. Other Health Outcomes

Further health outcomes are reported with seasonal variations in fatal health outcomes that are highest in winter (Table 1). Whether these risks are affected by the environmental factors identified above as those which have the greatest increases in mortality risks through CVD and respiratory illness (ambient temperature, atmospheric particulates, UVB production of vitamin D or other effects, or NO produced by UVA) is mostly unknown, though low 25(OH)D concentration has been considered a risk factor for each. A noteworthy feature of the diseases listed in this table is that only cancers have been found to have highly significant inverse correlations between incidence and mortality rates and solar UVB doses in mid-latitude countries [100], and it shows very little seasonal variation in mortality rates. Even though cancers develop slowly, serum 25(OH)D concentrations measured near the time of diagnosis are more strongly correlated with incidence than are concentrations measured more than a few months [111] or years [100] prior to diagnosis. Although RCTs have not found that supplementing with vitamin D reduces cancer incidence, it does reduce cancer mortality rates [101]. The failure of the many other RCTs in this area are likely attributable to poor design, conduct and analysis [100]. Thus, the lack of geographical ecological studies to support the role of vitamin D in reducing the risk of the other diseases already discussed supports the hypothesis that the non-vitamin-D-related effects of UVB exposure play important roles in reducing cancer and other health risks.

**Table 1:** Ratios of winter to summer mortality rate for common disorders in five countries.

Cause of Death	US 1951–1960 [112]	Australia 2015–2019 [113]	Japan 1970–1999 [114]	Netherlands 1979–1987 [115]	Scotland 1974–1988 [116]
All causes	1.17	1.10	1.04		1.33
Arteriosclerotic heart disease, CHD	1.28	1.15	1.14	1.34	1.28
Cancers	1.19	1.01	1.01	1.07	1.00
Cerebrovascular disease		1.11	1.09	1.25	1.30
Chronic respiratory disease		1.24		1.50	
Cirrhosis of liver	1.15				
Dementia		1.14			
Diabetes mellitus	1.10	1.12	1.20	1.28	
Digestive diseases			1.09		
Hypertensive heart disease	1.30				
Influenza				73	
Influenza, pneumonia (except newborn)	2.12	1.50	1.18		
Nephritis	1.22			1.30	
Nonrheumatic chronic endocarditis	1.24				
Pneumonia		1.33		1.88	
Respiratory					1.28
Rheumatic fever	1.21				
Septicemia				1.21	
Tuberculosis	1.17			1.59	
Vascular lesions, CNS	1.21				

CHD, coronary heart disease; CNS, central nervous system.

Both fever and high ambient temperatures are associated with exacerbations of multiple sclerosis and increased hospital admission rates [117,118]. Vitamin D deficiency is also associated with increased risks of MS, and the observational and epidemiological evidence for this association is increasingly supported by MR and mechanistic studies [119].

Type 1 diabetes results from autoimmune damage to islet beta cells that eventually destroys them. Variations occur in the risk of type 1 diabetes and of several other immune-mediated disorders with season of birth, which may be due to variations in either maternal or infant vitamin D status, in maternal or infant UVB exposure or both [120,121], but neither UVA exposure nor NO production has been investigated specifically as risk factors. Alhough vitamin D is necessary for normal insulin secretory responses to glucose and for maintaining healthy insulin sensitivity, it remains uncertain whether deficiency later in life increases type 1 diabetes risks [122].

Type 2 diabetes results from long periods of increased insulin resistance with high circulating insulin levels that lead to eventual islet beta cell failure. Since vitamin D is necessary for healthy insulin secretion and reduces abnormal insulin resistance through known mechanisms [123], vitamin D deficiency has long been thought likely to increase type 2 diabetes mellitus (T2DM) risks. That causal effect is supported by MR analyses [124] and by data from the Vitamin D and Type 2 Diabetes Study (D2d), showing up to 70% reductions in T2DM risk after 2.5 years of vitamin D supplementation of those subjects with prediabetes whose serum 25(OH)D values reached ≥100 nmol/l, a level only reached with intakes of 4000 IU/day [125].

Pregnancy-related disorders are more common in winter. Extremes of ambient temperature have adverse effects on birth outcomes, especially extremes of heat [126,127]. A recent literature review suggests that vitamin D deficiency has marked effects on pregnancy outcomes by increasing the risks of low birth weight, preterm birth and small-for-gestational-age births [128]. Deficiency is also associated with increased risks of gestational diabetes. Many trials of supplementation have failed to confirm causality in those disorders apart from gestational diabetes [129]. However, corrections of deficiency may require 4000 IU/day of vitamin D_3_ rather than the much smaller doses usually given in trials, and further work with adequate replacements in deficiency should clarify the situation [130].

### 2.7. Gene Expression

A pair of studies published in 2015 reported significant seasonal variations in gene expression, including the vitamin D receptor [131,132]. Several studies reported by Holick and colleagues also showed that vitamin D supplementation significantly affects gene expression [133,134,135]. Although none of those studies examined the role of the many genes modified in disease risk, many of vitamin D’s effects are due to controlling gene expression [136]. Since the risks of many diseases are lower in summer, when 25(OH)D concentrations are higher, it is reasonable to assume that genes with higher expression in summer are more likely to reduce the risk of disease. It is also likely that factors other than vitamin D affect seasonal variations in gene expression. For example, NO also regulates gene expression. By 2001, it was known that NO cannot only directly influence the activity of transcription factors, but it can also modulate upstream signaling cascades, mRNA stability and translation as well as the processing of the primary gene products [137]. A study published in 2003 reported that NO activates diverse signaling pathways to regulate gene expression experimentally [138].

### 2.8. Air Pollution

Air pollution is an important cause of mortality. Particulates smaller than 2.5 microns in diameter (PM_2.5_) were estimated to cause 4.2 million (95% CI, 3.7–4.8 million) deaths globally in 2015 [139]. PM_2.5_ is significantly correlated with chronic CHD mortality rates in the eastern U.S. during warm but not cold seasons [140]. Globally, 29% of the burden of stroke was attributed to air pollution in 2013 [141]. Air pollution is estimated to account for 790 thousand (95% CI, 645–934 thousand) deaths in Europe annually, with 40–80% from CVD [142]. A meta-analysis of the effects of air pollution on excess mortality rates reported in 2002 found that, for all common types of air pollutants, mortality rates were higher in warm seasons than in cold seasons [143]. It also found that excess mortality rates were about twice as high for respiratory diseases than for CVD for the pollutants, PM_10_, carbon monoxide, nitrogen dioxide and sulfates, but not for ozone.

## 3. Discussion

This narrative review indicates that seasonal variations in atmospheric temperature strongly affect the risk and severity of high BP, CVD and respiratory viral diseases. However, both solar-UVB-induced changes in 25(OH)D and UVA-induced changes in NO appear likely to contribute to seasonal variations in the risk of these disorders, albeit to a more limited extent. However, ensuring vitamin D repletion remains worthwhile for reducing mortality rates, especially for the elderly in winter.

More research is indicated to evaluate the relative contributions of each of the factors discussed to the risks of these diseases and to death rates. Since the environmental factors that were discussed as affecting CVD, BP and the two viral illnesses considered—ambient temperature and exposure to UVB and UVA—cannot easily be changed at the population level, observational studies remain important for further studies. However, data on relevant environmental and biological risk factors near to the time of disease development or of death may prove especially useful. Furthermore, the designs of both observational studies and of the RCTs of potentially protective measures for disorders with seasonal variations in risk need to provide for collecting data on ambient temperature, humidity, exposure to UVA and UVB radiation and on changes in vitamin D status and in NO production concurrently with other potentially relevant variables throughout all such studies.

Other common disorders whose risks vary with season, such as those of multiple sclerosis and pregnancy disorders, have not yet been investigated in enough detail to identify the contributions of variations in atmospheric temperature, UVB, UVA or humidity to those risks. However, the existing data on the contributions of the environmental factors discussed here to seasonal variations in mortality, CVD and respiratory virus infections already suggest that effective public health measures to avoid temperature and humidity extremes and to optimize exposure to UVB and UVA can improve public health. In the future, better information on the contributions of these, or other, environmental factors to other common disorders with seasonal variations in their presentations could also prove to help in developing public health measures that are able to reduce CVD and respiratory system risks. Public health measures for reducing CVD and respiratory system risks from seasonal variations in temperature and humidity can already be stated, from the information reviewed, to be important for reducing the mortality rates seen from CVD and respiratory disease. Furthermore, this importance increases with global warming, adding to the urgency of reducing this global hazard.

Because mortality rates are inversely correlated with solar UVB doses, the lack of adequate solar UVB can be compensated for by the use of suitably regulated indoor UVB lamps. Studies conducted in England indicate that using a subliminal UVB source delivering the equivalent of 15 min of summer sunshine significantly increases the 25(OH)D concentrations of elderly residents of a residential of care homes [144,145]. The authors recommended additional studies before this approach could be used to recommend changes in health policy. UV fluorescent lamps have, however, already been used to increase vitamin D [146,147]. More recently, UVB sources using LEDs are being developed for stimulating vitamin D_3_ production [148,149].

African Americans and other dark-skinned people living at higher latitudes than those of their ancestral homelands generally have poorer health outcomes than fair-skinned people in the same locations [150]. Although that article recommended vitamin D supplementation to help reduce ethnic health disparities, it now appears that UVB exposure should be considered as well.

Sunscreen can block UVB radiation from penetrating the skin. Since mounting evidence indicates that UVB has health benefits beyond vitamin D production, people should consider spending 15–30 min in the midday summer sun without sunscreen. Doing so daily can result in protective tanning in most, but not for all white subjects [151]. The protection from tanning is equivalent to a sun protection factor of 3–4 [152], meaning that one can stay in the sun 3–4 times longer without erythema than without a tan. In addition, raising serum 25(OH)D concentrations above 40 ng/mL can reduce the production of erythema from solar UVB exposure [153,154].

A recent article furnished data to help guide medical recommendations for sensible sun exposure to promote the cutaneous production of vitamin D_3_ at different latitudes, seasonality, time of day and cloudiness status in Brazil [155] and these guidelines should be applicable elsewhere as well.

Hopefully, additional research can provide more information on the role of solar UVA and UVB in reducing the risks of seasonally variable diseases and mortality, leading to improved public health and clinical practice guidelines. Meanwhile, habitual increases in exposure to summer sunshine [with avoidance of sunburn] would also contribute to improving the public health [154].

## References

[1] Marti-Soler H., Gonseth S., Gubelmann C., Stringhini S., Bovet P., Chen P.C., Wojtyniak B., Paccaud F., Tsai D.H., Zdrojewski T. (2014). Seasonal variation of overall and cardiovascular mortality: A study in 19 countries from different geographic locations. PLoS ONE.

[2] Bunker A., Wildenhain J., Vandenbergh A., Henschke N., Rocklov J., Hajat S., Sauerborn R. (2016). Effects of Air Temperature on Climate-Sensitive Mortality and Morbidity Outcomes in the Elderly; a Systematic Review and Meta-analysis of Epidemiological Evidence. EBioMedicine.

[3] Burkart K.G., Brauer M., Aravkin A.Y., Godwin W.W., Hay S.I., He J., Iannucci V.C., Larson S.L., Lim S.S., Liu J. (2021). Estimating the cause-specific relative risks of non-optimal temperature on daily mortality: A two-part modelling approach applied to the Global Burden of Disease Study. Lancet.

[4] Baggerly C.A., Cuomo R.E., French C.B., Garland C.F., Gorham E.D., Grant W.B., Heaney R.P., Holick M.F., Hollis B.W., McDonnell S.L. (2015). Sunlight and Vitamin D: Necessary for Public Health. J. Am. Coll. Nutr..

[5] Holick M.F. (2016). Biological Effects of Sunlight, Ultraviolet Radiation, Visible Light, Infrared Radiation and Vitamin D for Health. Anticancer Res..

[6] Grant W.B., Bhattoa H.P., Boucher B.J. (2017). Seasonal variations of U.S. mortality rates: Roles of solar ultraviolet-B doses, vitamin D, gene expression, and infections. J. Steroid Biochem. Mol. Biol..

[7] Feelisch M., Kolb-Bachofen V., Liu D., Lundberg J.O., Revelo L.P., Suschek C.V., Weller R.B. (2010). Is sunlight good for our heart?. Eur. Heart J..

[8] Liu D., Fernandez B.O., Hamilton A., Lang N.N., Gallagher J.M.C., Newby D.E., Feelisch M., Weller R.B. (2014). UVA irradiation of human skin vasodilates arterial vasculature and lowers blood pressure independently of nitric oxide synthase. J. Investig. Dermatol..

[9] Cherrie M., Clemens T., Colandrea C., Feng Z., Webb D.J., Weller R.B., Dibben C. (2021). Ultraviolet A radiation and COVID-19 deaths in the USA with replication studies in England and Italy. Br. J. Dermatol..

[10] Grant W.B., Bhattoa H.P., Pludowski P., Feldman D., Pike J.W. (2022). Determinants of Vitamin D Levels from Sun Exposure. Vitamin D, Ch. 56.

[11] Engelsen O. (2010). The relationship between ultraviolet radiation exposure and vitamin D status. Nutrients.

[12] Grant W.B., Fakhoury H.M.A., Karras S.N., Al Anouti F., Bhattoa H.P. (2019). Variations in 25-Hydroxyvitamin D in Countries from the Middle East and Europe: The Roles of UVB Exposure and Diet. Nutrients.

[13] Scragg R. (1981). Seasonality of cardiovascular disease mortality and the possible protective effect of ultra-violet radiation. Int. J. Epidemiol..

[14] Rostand S.G. (1997). Ultraviolet light may contribute to geographic and racial blood pressure differences. Hypertension.

[15] Ponsonby A.L., McMichael A., van der Mei I. (2002). Ultraviolet radiation and autoimmune disease: Insights from epidemiological research. Toxicology.

[16] Cannell J.J., Vieth R., Umhau J.C., Holick M.F., Grant W.B., Madronich S., Garland C.F., Giovannucci E. (2006). Epidemic influenza and vitamin D. Epidemiol. Infect..

[17] Lindqvist P.G., Olsson H., Landin-Olsson M. (2010). Are active sun exposure habits related to lowering risk of type 2 diabetes mellitus in women, a prospective cohort study?. Diabetes Res. Clin. Pract..

[18] Lindqvist P.G., Epstein E., Olsson H. (2009). Does an active sun exposure habit lower the risk of venous thrombotic events? A D-lightful hypothesis. J. Thromb. Haemost..

[19] Lindqvist P.G., Epstein E., Nielsen K., Landin-Olsson M., Ingvar C., Olsson H. (2016). Avoidance of sun exposure as a risk factor for major causes of death: A competing risk analysis of the Melanoma in Southern Sweden cohort. J. Intern. Med..

[20] Lindqvist P.G., Epstein E., Landin-Olsson M. (2022). Sun Exposure—Hazards and Benefits. Anticancer Res..

[21] Webb A.R., Kline L., Holick M.F. (1988). Influence of season and latitude on the cutaneous synthesis of vitamin D3: Exposure to winter sunlight in Boston and Edmonton will not promote vitamin D3 synthesis in human skin. J. Clin. Endocrinol. Metab..

[22] Hypponen E., Power C. (2007). Hypovitaminosis D in British adults at age 45 y: Nationwide cohort study of dietary and lifestyle predictors. Am. J. Clin. Nutr..

[23] Kroll M.H., Bi C., Garber C.C., Kaufman H.W., Liu D., Caston-Balderrama A., Zhang K., Clarke N., Xie M., Reitz R.E. (2015). Temporal relationship between vitamin D status and parathyroid hormone in the United States. PLoS ONE.

[24] Mason R.S., Rybchyn M.S., Abboud M., Brennan-Speranza T.C., Fraser D.R. (2019). The Role of Skeletal Muscle in Maintaining Vitamin D Status in Winter. Curr. Dev. Nutr..

[25] Rybchyn M.S., Abboud M., Puglisi D.A., Gordon-Thomson C., Brennan-Speranza T.C., Mason R.S., Fraser D.R. (2020). Skeletal Muscle and the Maintenance of Vitamin D Status. Nutrients.

[26] Katsuki S., Arnold W., Mittal C., Murad F. (1977). Stimulation of guanylate cyclase by sodium nitroprusside, nitroglycerin and nitric oxide in various tissue preparations and comparison to the effects of sodium azide and hydroxylamine. J. Cycl. Nucleotide Res..

[27] Gruetter C.A., Barry B.K., McNamara D.B., Gruetter D.Y., Kadowitz P.J., Ignarro L. (1979). Relaxation of bovine coronary artery and activation of coronary arterial guanylate cyclase by nitric oxide, nitroprusside and a carcinogenic nitrosoamine. J. Cycl. Nucleotide Res..

[28] Furchgott R.F., Zawadzki J.V. (1980). The obligatory role of endothelial cells in the relaxation of arterial smooth muscle by acetylcholine. Nature.

[29] SoRelle R. (1998). Nobel prize awarded to scientists for nitric oxide discoveries. Circulation.

[30] Ignarro L.J. (2019). Nitric oxide is not just blowing in the wind. Br. J. Pharmacol..

[31] Weller R. (1997). Nitric oxide—A newly discovered chemical transmitter in human skin. Br. J. Dermatol..

[32] Oplander C., Volkmar C.M., Paunel-Gorgulu A., van Faassen E.E., Heiss C., Kelm M., Halmer D., Murtz M., Pallua N., Suschek C.V. (2009). Whole body UVA irradiation lowers systemic blood pressure by release of nitric oxide from intracutaneous photolabile nitric oxide derivates. Circ. Res..

[33] Juzeniene A., Brekke P., Dahlback A., Andersson-Engels S., Reichrath J., Moan K., Holick M.F., Grant W.B., Moan J. (2011). Solar radiation and human health. Rep. Prog. Phys..

[34] Juzeniene A., Moan J. (2012). Beneficial effects of UV radiation other than via vitamin D production. Derm. Endocrinol..

[35] Muggeridge D.J., Sculthorpe N., Grace F.M., Willis G., Thornhill L., Weller R.B., James P.E., Easton C. (2015). Acute whole body UVA irradiation combined with nitrate ingestion enhances time trial performance in trained cyclists. Nitric Oxide.

[36] Gorman S., Black L.J., Feelisch M., Hart P.H., Weller R. (2015). Can skin exposure to sunlight prevent liver inflammation?. Nutrients.

[37] Fleury N., Feelisch M., Hart P.H., Weller R.B., Smoothy J., Matthews V.B., Gorman S. (2017). Sub-erythemal ultraviolet radiation reduces metabolic dysfunction in already overweight mice. J. Endocrinol..

[38] Yu B., Jin S., Wang C., Yan S., Zhou X., Cui X., Tang Z., Luan Q., Guo Y., Bian Z. (2020). The association of outdoor temperature with blood pressure, and its influence on future cardio-cerebrovascular disease risk in cold areas. J. Hypertens..

[39] Hu J., He G., Luo J., Xu Y., Xu X., Song X., Chen S., Ji G., Chen Z., Jiang Q. (2021). Temperature-adjusted hypertension prevalence and control rate: A series of cross-sectional studies in Guangdong Province, China. J. Hypertens..

[40] Group I.C.R. (1988). Intersalt: An international study of electrolyte excretion and blood pressure. Results for 24 hour urinary sodium and potassium excretion. Intersalt Cooperative Research Group. BMJ.

[41] Rostand S.G., McClure L.A., Kent S.T., Judd S.E., Gutierrez O.M. (2016). Associations of blood pressure, sunlight, and vitamin D in community-dwelling adults. J. Hypertens..

[42] Warren J.B. (1994). Nitric oxide and human skin blood flow responses to acetylcholine and ultraviolet light. FASEB J..

[43] Andrukhova O., Slavic S., Zeitz U., Riesen S.C., Heppelmann M.S., Ambrisko T.D., Markovic M., Kuebler W.M., Erben R.G. (2014). Vitamin D is a regulator of endothelial nitric oxide synthase and arterial stiffness in mice. Mol. Endocrinol..

[44] Weller R.B., Wang Y., He J., Maddux F.W., Usvyat L., Zhang H., Feelisch M., Kotanko P. (2020). Does Incident Solar Ultraviolet Radiation Lower Blood Pressure?. J. Am. Heart Assoc..

[45] Weller R.B., Feelisch M., Kotanko P. (2020). Correspondence on ‘Seasonal variation in blood pressure: Evidence, consensus and recommendations for clinical practice. Consensus statement by the ESH Working Group on Blood Pressure Monitoring and Cardiovascular Variability’. J. Hypertens..

[46] Zhou A., Selvanayagam J.B., Hypponen E. (2021). Non-linear Mendelian randomization analyses support a role for vitamin D deficiency in cardiovascular disease risk. Eur. Heart J..

[47] Mirhosseini N., Vatanparast H., Kimball S.M. (2017). The Association between Serum 25(OH)D Status and Blood Pressure in Participants of a Community-Based Program Taking Vitamin D Supplements. Nutrients.

[48] Kutschenreuter P.H. (1959). A study of the effect of weather on mortality. Trans. N. Y. Acad. Sci..

[49] Wilkinson P., Pattenden S., Armstrong B., Fletcher A., Kovats R.S., Mangtani P., McMichael A.J. (2004). Vulnerability to winter mortality in elderly people in Britain: Population based study. BMJ.

[50] Garland C.F., Kim J.J., Mohr S.B., Gorham E.D., Grant W.B., Giovannucci E.L., Baggerly L., Hofflich H., Ramsdell J.W., Zeng K. (2014). Meta-analysis of all-cause mortality according to serum 25-hydroxyvitamin D. Am. J. Public Health.

[51] Revich B., Shaposhnikov D. (2008). Temperature-induced excess mortality in Moscow, Russia. Int. J. Biometeorol..

[52] Donaldson G.C., Ermakov S.P., Komarov Y.M., McDonald C.P., Keatinge W.R. (1998). Cold related mortalities and protection against cold in Yakutsk, eastern Siberia: Observation and interview study. BMJ.

[53] Keatinge W.R., Coleshaw S.R., Holmes J. (1989). Changes in seasonal mortalities with improvement in home heating in England and Wales from 1964 to 1984. Int. J. Biometeorol..

[54] Achebak H., Devolder D., Ballester J. (2019). Trends in temperature-related age-specific and sex-specific mortality from cardiovascular diseases in Spain: A national time-series analysis. Lancet Planet. Health.

[55] Keatinge W.R. (2002). Winter mortality and its causes. Int. J. Circumpolar Health.

[56] Kenney W.L., Munce T.A. (2003). Invited review: Aging and human temperature regulation. J. Appl. Physiol..

[57] Grimes D.S., Hindle E., Dyer T. (1996). Sunlight, cholesterol and coronary heart disease. QJM.

[58] Zittermann A., Schleithoff S.S., Koerfer R. (2005). Putting cardiovascular disease and vitamin D insufficiency into perspective. Br. J. Nutr..

[59] Thurston G.D., Burnett R.T., Turner M.C., Shi Y., Krewski D., Lall R., Ito K., Jerrett M., Gapstur S.M., Diver W.R. (2016). Ischemic Heart Disease Mortality and Long-Term Exposure to Source-Related Components of U.S. Fine Particle Air Pollution. Environ. Health Perspect..

[60] Acharya P., Dalia T., Ranka S., Sethi P., Oni O.A., Safarova M.S., Parashara D., Gupta K., Barua R.S. (2021). The Effects of Vitamin D Supplementation and 25-Hydroxyvitamin D Levels on the Risk of Myocardial Infarction and Mortality. J. Endocr. Soc..

[61] Manson J.E., Cook N.R., Lee I.M., Christen W., Bassuk S.S., Mora S., Gibson H., Gordon D., Copeland T., D’Agostino D. (2019). Vitamin D Supplements and Prevention of Cancer and Cardiovascular Disease. N. Engl. J. Med..

[62] Barbarawi M., Kheiri B., Zayed Y., Barbarawi O., Dhillon H., Swaid B., Yelangi A., Sundus S., Bachuwa G., Alkotob M.L. (2019). Vitamin D Supplementation and Cardiovascular Disease Risks in More Than 83000 Individuals in 21 Randomized Clinical Trials: A Meta-analysis. JAMA Cardiol..

[63] Raed A., Bhagatwala J., Zhu H., Pollock N.K., Parikh S.J., Huang Y., Havens R., Kotak I., Guo D.H., Dong Y. (2017). Dose responses of vitamin D3 supplementation on arterial stiffness in overweight African Americans with vitamin D deficiency: A placebo controlled randomized trial. PLoS ONE.

[64] Emerging Risk Factors Collaboration/EPIC-CVD/Vitamin D Studies Collaboration (2021). Estimating dose-response relationships for vitamin D with coronary heart disease, stroke, and all-cause mortality: Observational and Mendelian randomisation analyses. Lancet Diabetes Endocrinol..

[65] Heaney R.P. (2014). Design and analysis of clinical trials of nutrients: Author reply. Nutr. Rev..

[66] Grant W.B., Boucher B.J., Bhattoa H.P., Lahore H. (2018). Why vitamin D clinical trials should be based on 25-hydroxyvitamin D concentrations. J. Steroid Biochem. Mol. Biol..

[67] Anderson J.L., Vanwoerkom R.C., Horne B.D., Bair T.L., May H.T., Lappe D.L., Muhlestein J.B. (2011). Parathyroid hormone, vitamin D, renal dysfunction, and cardiovascular disease: Dependent or independent risk factors?. Am. Heart J..

[68] Valcour A., Blocki F., Hawkins D.M., Rao S.D. (2012). Effects of age and serum 25-OH-vitamin D on serum parathyroid hormone levels. J. Clin. Endocrinol. Metab..

[69] Tomaschitz A., Ritz E., Pieske B., Rus-Machan J., Kienreich K., Verheyen N., Gaksch M., Grubler M., Fahrleitner-Pammer A., Mrak P. (2014). Aldosterone and parathyroid hormone interactions as mediators of metabolic and cardiovascular disease. Metabolism.

[70] Bolland M.J., Avenell A., Baron J.A., Grey A., MacLennan G.S., Gamble G.D., Reid I.R. (2010). Effect of calcium supplements on risk of myocardial infarction and cardiovascular events: Meta-analysis. BMJ.

[71] Boucher B.J. (2012). Calcium supplements may increase the risk of cardiovascular events in postmenopausal women. Evid. Based Med..

[72] Lei J., Vodovotz Y., Tzeng E., Billiar T.R. (2013). Nitric oxide, a protective molecule in the cardiovascular system. Nitric Oxide.

[73] Weller R.B. (2016). Sunlight Has Cardiovascular Benefits Independently of Vitamin D. Blood Purif..

[74] Fuchs F.D., Whelton P.K. (2020). High Blood Pressure and Cardiovascular Disease. Hypertension.

[75] Mackay D.F., Clemens T.L., Hastie C.E., Cherrie M.P.C., Dibben C., Pell J.P. (2019). UVA and Seasonal Patterning of 56 370 Myocardial Infarctions Across Scotland, 2000–2011. J. Am. Heart Assoc..

[76] Bhatnagar A. (2017). Environmental Determinants of Cardiovascular Disease. Circ. Res..

[77] Hope-Simpson R.E. (1981). The role of season in the epidemiology of influenza. J. Hyg..

[78] Aloia J.F., Li-Ng M. (2007). Re: Epidemic influenza and vitamin D. Epidemiol. Infect..

[79] Li-Ng M., Aloia J.F., Pollack S., Cunha B.A., Mikhail M., Yeh J., Berbari N. (2009). A randomized controlled trial of vitamin D3 supplementation for the prevention of symptomatic upper respiratory tract infections. Epidemiol. Infect..

[80] Urashima M., Segawa T., Okazaki M., Kurihara M., Wada Y., Ida H. (2010). Randomized trial of vitamin D supplementation to prevent seasonal influenza A in schoolchildren. Am. J. Clin. Nutr..

[81] Shaman J., Jeon C.Y., Giovannucci E., Lipsitch M. (2011). Shortcomings of vitamin D-based model simulations of seasonal influenza. PLoS ONE.

[82] Tamerius J.D., Shaman J., Alonso W.J., Bloom-Feshbach K., Uejio C.K., Comrie A., Viboud C. (2013). Environmental predictors of seasonal influenza epidemics across temperate and tropical climates. PLoS Pathog..

[83] Ianevski A., Zusinaite E., Shtaida N., Kallio-Kokko H., Valkonen M., Kantele A., Telling K., Lutsar I., Letjuka P., Metelitsa N. (2019). Low Temperature and Low UV Indexes Correlated with Peaks of Influenza Virus Activity in Northern Europe during 2010–2018. Viruses.

[84] Harper G.J. (1961). Airborne micro-organisms: Survival tests with four viruses. J. Hyg..

[85] Audi A., AlIbrahim M., Kaddoura M., Hijazi G., Yassine H.M., Zaraket H. (2020). Seasonality of Respiratory Viral Infections: Will COVID-19 Follow Suit?. Front. Public Health.

[86] Eccles R. (2002). An explanation for the seasonality of acute upper respiratory tract viral infections. Acta Otolaryngol..

[87] Khalis M., Toure A.B., El Badisy I., Khomsi K., Najmi H., Bouaddi O., Marfak A., Al-Delaimy W.K., Berraho M., Nejjari C. (2022). Relationship between Meteorological and Air Quality Parameters and COVID-19 in Casablanca Region, Morocco. Int. J. Environ. Res. Public Health.

[88] Grant W.B., Lahore H., McDonnell S.L., Baggerly C.A., French C.B., Aliano J.L., Bhattoa H.P. (2020). Evidence that Vitamin D Supplementation Could Reduce Risk of Influenza and COVID-19 Infections and Deaths. Nutrients.

[89] Oristrell J., Oliva J.C., Casado E., Subirana I., Dominguez D., Toloba A., Balado A., Grau M. (2022). Vitamin D supplementation and COVID-19 risk: A population-based, cohort study. J. Endocrinol. Investig..

[90] Seal K.H., Bertenthal D., Carey E., Grunfeld C., Bikle D.D., Lu C.M. (2022). Association of Vitamin D Status and COVID-19-Related Hospitalization and Mortality. J. Gen. Intern. Med..

[91] Entrenas Castillo M., Entrenas Costa L.M., Vaquero Barrios J.M., Alcala Diaz J.F., Lopez Miranda J., Bouillon R., Quesada Gomez J.M. (2020). Effect of calcifediol treatment and best available therapy versus best available therapy on intensive care unit admission and mortality among patients hospitalized for COVID-19: A pilot randomized clinical study. J. Steroid Biochem. Mol. Biol..

[92] Villasis-Keever M.A., Lopez-Alarcon M.G., Miranda-Vovales G., Zurita-Cruz J.N., Barrada-Vazquez A.Z. (2022). Efficacy and Safety of Vitamin D Supplementation to Prevent COVID-19 in Frontline Healthcare Workers. A Randomized Clinical Trial. Arch. Med. Res..

[93] Jenkins C.R., Celli B., Anderson J.A., Ferguson G.T., Jones P.W., Vestbo J., Yates J.C., Calverley P.M. (2012). Seasonality and determinants of moderate and severe COPD exacerbations in the TORCH study. Eur. Respir. J..

[94] Donaldson G.C., Wedzicha J.A. (2014). The causes and consequences of seasonal variation in COPD exacerbations. Int. J. Chron. Obstruct. Pulmon. Dis..

[95] Guillot X., Semerano L., Saidenberg-Kermanac’h N., Falgarone G., Boissier M.C. (2010). Vitamin D and inflammation. Jt. Bone Spine.

[96] Ma W., Nguyen L.H., Yue Y., Ding M., Drew D.A., Wang K., Merino J., Rich-Edwards J.W., Sun Q., Camargo C.A. (2022). Associations between predicted vitamin D status, vitamin D intake, and risk of severe acute respiratory syndrome coronavirus 2 (SARS-CoV-2) infection and coronavirus disease 2019 (COVID-19) severity. Am. J. Clin. Nutr..

[97] Lau F.H., Powell C.E., Adonecchi G., Danos D.M., DiNardo A.R., Chugden R.J., Wolr P., Castilla C.F. (2022). Pilot Phase Results of a Prospective, Randomized Controlled Trial of Narrowband Ultraviolet B Phototherapy in Hospitalized COVID-19 Patients. Exp. Dermatol..

[98] Walrand S. (2021). Autumn COVID-19 surge dates in Europe correlated to latitudes, not to temperature-humidity, pointing to vitamin D as contributing factor. Sci. Rep..

[99] Gorman S., Weller R.B. (2020). Investigating the Potential for Ultraviolet Light to Modulate Morbidity and Mortality From COVID-19: A Narrative Review and Update. Front. Cardiovasc. Med..

[100] Muñoz A., Grant W.B. (2022). Vitamin D and Cancer: An Historical Overview of the Epidemiology and Mechanisms. Nutrients.

[101] Keum N., Lee D.H., Greenwood D.C., Manson J.E., Giovannucci E. (2019). Vitamin D supplementation and total cancer incidence and mortality: A meta-analysis of randomized controlled trials. Ann. Oncol..

[102] Sharma A., Sharma T., Panwar M.S., Sharma D., Bundel R., Hamilton R.T., Radosevich J.A., Mandal C.C. (2017). Colder environments are associated with a greater cancer incidence in the female population of the United States. Tumour Biol..

[103] Bandyopadhayaya S., Ford B., Mandal C.C. (2020). Cold-hearted: A case for cold stress in cancer risk. J. Therm. Biol..

[104] Lu J., Wei Q., Bondy M.L., Yu T.K., Li D., Brewster A., Shete S., Sahin A., Meric-Bernstam F., Wang L.E. (2006). Promoter polymorphism (-786t>C) in the endothelial nitric oxide synthase gene is associated with risk of sporadic breast cancer in non-Hispanic white women age younger than 55 years. Cancer.

[105] Lee K.M., Kang D., Park S.K., Berndt S.I., Reding D., Chatterjee N., Chanock S., Huang W.Y., Hayes R.B. (2009). Nitric oxide synthase gene polymorphisms and prostate cancer risk. Carcinogenesis.

[106] Goto T., Haruma K., Kitadai Y., Ito M., Yoshihara M., Sumii K., Hayakawa N., Kajiyama G. (1999). Enhanced expression of inducible nitric oxide synthase and nitrotyrosine in gastric mucosa of gastric cancer patients. Clin. Cancer Res..

[107] Mason B.H., Holdaway I.M., Stewart A.W., Neave L.M., Kay R.G. (1990). Season of tumour detection influences factors predicting survival of patients with breast cancer. Breast Cancer Res. Treat..

[108] Porojnicu A.C., Dahlback A., Moan J. (2008). Sun exposure and cancer survival in Norway: Changes in the risk of death with season of diagnosis and latitude. Adv. Exp. Med. Biol..

[109] Chandler P.D., Chen W.Y., Ajala O.N., Hazra A., Cook N., Bubes V., Lee I.M., Giovannucci E.L., Willett W., Buring J.E. (2020). Effect of Vitamin D3 Supplements on Development of Advanced Cancer: A Secondary Analysis of the VITAL Randomized Clinical Trial. JAMA Netw. Open.

[110] Madden J.M., Murphy L., Zgaga L., Bennett K. (2018). De novo vitamin D supplement use post-diagnosis is associated with breast cancer survival. Breast Cancer Res. Treat..

[111] Mohr S.B., Gorham E.D., Alcaraz J.E., Kane C.I., Macera C.A., Parsons J.K., Wingard D.L., Horst R., Garland C.F. (2013). Serum 25-hydroxyvitamin D and breast cancer in the military: A case-control study utilizing pre-diagnostic serum. Cancer Causes Control..

[112] Rosenwaike I. (1966). Seasonal variation of deaths in the United States, 1951–1960. J. Am. Stat. Assoc..

[113] Gregory G., Zhu L., Hayen A., Bell K.J.L. (2022). Learning from the pandemic: Mortality trends and seasonality of deaths in Australia in 2020. Int. J. Epidemiol..

[114] Nakaji S., Parodi S., Fontana V., Umeda T., Suzuki K., Sakamoto J., Fukuda S., Wada S., Sugawara K. (2004). Seasonal changes in mortality rates from main causes of death in Japan (1970–1999). Eur. J. Epidemiol..

[115] Mackenbach J.P., Kunst A.E., Looman C.W. (1992). Seasonal variation in mortality in The Netherlands. J. Epidemiol. Community Health.

[116] Douglas A.S., Allan T.M., Rawles J.M. (1991). Composition of seasonality of disease. Scott. Med. J..

[117] Ebi K.L., Capon A., Berry P., Broderick C., de Dear R., Havenith G., Honda Y., Kovats R.S., Ma W., Malik A. (2021). Hot weather and heat extremes: Health risks. Lancet.

[118] Vandebergh M., Degryse N., Dubois B., Goris A. (2022). Environmental risk factors in multiple sclerosis: Bridging Mendelian randomization and observational studies. J. Neurol..

[119] Wang R. (2022). Mendelian randomization study updates the effect of 25-hydroxyvitamin D levels on the risk of multiple sclerosis. J. Transl. Med..

[120] Sloka S., Grant M., Newhook L.A. (2010). The geospatial relation between UV solar radiation and type 1 diabetes in Newfoundland. Acta Diabetol..

[121] Disanto G., Chaplin G., Morahan J.M., Giovannoni G., Hypponen E., Ebers G.C., Ramagopalan S.V. (2012). Month of birth, vitamin D and risk of immune-mediated disease: A case control study. BMC Med..

[122] Boucher B.J., Mannan N., Noonan K., Hales C.N., Evans S.J. (1995). Glucose intolerance and impairment of insulin secretion in relation to vitamin D deficiency in east London Asians. Diabetologia.

[123] Niroomand M., Fotouhi A., Irannejad N., Hosseinpanah F. (2019). Does high-dose vitamin D supplementation impact insulin resistance and risk of development of diabetes in patients with pre-diabetes? A double-blind randomized controlled trial. Diabetes Res. Clin. Pract..

[124] Bejar C.A., Goyal S., Afzal S., Mangino M., Zhou A., van der Most P.J., Bao Y., Gupta V., Smart M.C., Walia G.K. (2021). A Bidirectional Mendelian Randomization Study to evaluate the causal role of reduced blood vitamin D levels with type 2 diabetes risk in South Asians and Europeans. Nutr. J..

[125] Dawson-Hughes B., Staten M.A., Knowler W.C., Nelson J., Vickery E.M., LeBlanc E.S., Neff L.M., Park J., Pittas A.G., Group D.d.R. (2020). Intratrial Exposure to Vitamin D and New-Onset Diabetes Among Adults With Prediabetes: A Secondary Analysis From the Vitamin D and Type 2 Diabetes (D2d) Study. Diabetes Care.

[126] Zhang Y., Yu C., Wang L. (2017). Temperature exposure during pregnancy and birth outcomes: An updated systematic review of epidemiological evidence. Environ. Pollut..

[127] Samuels L., Nakstad B., Roos N., Bonell A., Chersich M., Havenith G., Luchters S., Day L.T., Hirst J.E., Singh T. (2022). Physiological mechanisms of the impact of heat during pregnancy and the clinical implications: Review of the evidence from an expert group meeting. Int. J. Biometeorol..

[128] Zhao R., Zhou L., Wang S., Yin H., Yang X., Hao L. (2022). Effect of maternal vitamin D status on risk of adverse birth outcomes: A systematic review and dose-response meta-analysis of observational studies. Eur. J. Nutr..

[129] Nausheen S., Habib A., Bhura M., Rizvi A., Shaheen F., Begum K., Iqbal J., Ariff S., Shaikh L., Raza S.S. (2021). Impact evaluation of the efficacy of different doses of vitamin D supplementation during pregnancy on pregnancy and birth outcomes: A randomised, controlled, dose comparison trial in Pakistan. BMJ Nutr. Prev. Health.

[130] Wagner C.L., Hollis B.W. (2018). The Implications of Vitamin D Status During Pregnancy on Mother and her Developing Child. Front. Endocrinol..

[131] Dopico X.C., Evangelou M., Ferreira R.C., Guo H., Pekalski M.L., Smyth D.J., Cooper N., Burren O.S., Fulford A.J., Hennig B.J. (2015). Widespread seasonal gene expression reveals annual differences in human immunity and physiology. Nat. Commun..

[132] Goldinger A., Shakhbazov K., Henders A.K., McRae A.F., Montgomery G.W., Powell J.E. (2015). Seasonal effects on gene expression. PLoS ONE.

[133] Hossein-nezhad A., Spira A., Holick M.F. (2013). Influence of vitamin D status and vitamin D3 supplementation on genome wide expression of white blood cells: A randomized double-blind clinical trial. PLoS ONE.

[134] Shirvani A., Kalajian T.A., Song A., Holick M.F. (2019). Disassociation of Vitamin D’s Calcemic Activity and Non-calcemic Genomic Activity and Individual Responsiveness: A Randomized Controlled Double-Blind Clinical Trial. Sci. Rep..

[135] Shirvani A., Kalajian T.A., Song A., Allen R., Charoenngam N., Lewanczuk R., Holick M.F. (2020). Variable Genomic and Metabolomic Responses to Varying Doses of Vitamin D Supplementation. Anticancer Res..

[136] Wimalawansa S.J. (2019). Vitamin D Deficiency: Effects on Oxidative Stress, Epigenetics, Gene Regulation, and Aging. Biology.

[137] Bogdan C. (2001). Nitric oxide and the regulation of gene expression. Trends Cell Biol..

[138] Hemish J., Nakaya N., Mittal V., Enikolopov G. (2003). Nitric oxide activates diverse signaling pathways to regulate gene expression. J. Biol. Chem..

[139] Cohen A.J., Brauer M., Burnett R., Anderson H.R., Frostad J., Estep K., Balakrishnan K., Brunekreef B., Dandona L., Dandona R. (2017). Estimates and 25-year trends of the global burden of disease attributable to ambient air pollution: An analysis of data from the Global Burden of Diseases Study 2015. Lancet.

[140] Hu Z. (2009). Spatial analysis of MODIS aerosol optical depth, PM2.5, and chronic coronary heart disease. Int. J. Health Geogr..

[141] Feigin V.L., Roth G.A., Naghavi M., Parmar P., Krishnamurthi R., Chugh S., Mensah G.A., Norrving B., Shiue I., Ng M. (2016). Global burden of stroke and risk factors in 188 countries, during 1990–2013: A systematic analysis for the Global Burden of Disease Study 2013. Lancet Neurol..

[142] Lelieveld J., Klingmuller K., Pozzer A., Poschl U., Fnais M., Daiber A., Munzel T. (2019). Cardiovascular disease burden from ambient air pollution in Europe reassessed using novel hazard ratio functions. Eur. Heart J..

[143] Stieb D.M., Judek S., Burnett R.T. (2002). Meta-analysis of time-series studies of air pollution and mortality: Effects of gases and particles and the influence of cause of death, age, and season. J. Air Waste Manag. Assoc..

[144] Corless D., Gupta S.P., Switala S., Barragry J.M., Boucher B.J., Cohen R.D., Diffey B.L. (1978). Response of plasma-25-hydroxyvitamin D to ultraviolet irradiation in long-stay geriatric patients. Lancet.

[145] Chuck A., Todd J., Diffey B. (2001). Subliminal ultraviolet-B irradiation for the prevention of vitamin D deficiency in the elderly: A feasibility study. Photodermatol. Photoimmunol. Photomed..

[146] Chandra P., Wolfenden L.L., Ziegler T.R., Tian J., Luo M., Stecenko A.A., Chen T.C., Holick M.F., Tangpricha V. (2007). Treatment of vitamin D deficiency with UV light in patients with malabsorption syndromes: A case series. Photodermatol. Photoimmunol. Photomed..

[147] Dabai N.S., Pramyothin P., Holick M.F. (2012). The effect of ultraviolet radiation from a novel portable fluorescent lamp on serum 25-hydroxyvitamin D3 levels in healthy adults with Fitzpatrick skin types II and III. Photodermatol. Photoimmunol. Photomed..

[148] Park D.-H., Oh S.-T., Lim J.-H. (2019). Development of UVB LED Lighting System Based on UV Dose Calculation Algorithm to Meet Individual Daily UV Dose. Appl. Sci..

[149] Oh S.Y., Lim J.-H. (2020). Development and Effect Analysis of UVB-LED General Lighting to Support Vitamin D Synthesis. Appl. Sci..

[150] Ames B.N., Grant W.B., Willett W.C. (2021). Does the High Prevalence of Vitamin D Deficiency in African Americans Contribute to Health Disparities?. Nutrients.

[151] Jablonski N.G., Chaplin G. (2010). Colloquium paper: Human skin pigmentation as an adaptation to UV radiation. Proc. Natl. Acad. Sci. USA.

[152] Mohania D., Chandel S., Kumar P., Verma V., Digvijay K., Tripathi D., Choudhury K., Mitten S.K., Shah D. (2017). Ultraviolet Radiations: Skin Defense-Damage Mechanism. Adv. Exp. Med. Biol..

[153] Scott J.F., Das L.M., Ahsanuddin S., Qiu Y., Binko A.M., Traylor Z.P., Debanne S.M., Cooper K.D., Boxer R., Lu K.Q. (2017). Oral Vitamin D Rapidly Attenuates Inflammation from Sunburn: An Interventional Study. J. Investig. Dermatol..

[154] Scott J.F., Lu K.Q. (2017). Vitamin D as a Therapeutic Option for Sunburn: Clinical and Biologic Implications. DNA Cell Biol..

[155] Leal A., Correa M.P., Holick M.F., Melo E.V., Lazaretti-Castro M. (2021). Sun-induced production of vitamin D3 throughout 1 year in tropical and subtropical regions: Relationship with latitude, cloudiness, UV-B exposure and solar zenith angle. Photochem. Photobiol. Sci..

[156] Reichrath J., Vogt T., Holick M.F., Friedrich M. (2022). Abstracts of the Joint International Symposia “Vitamin D in Prevention and Therapy” and “Biologic Effects of Light”. Anticancer Res..

